# Research on health equity in the SDG era: the urgent need for greater focus on implementation

**DOI:** 10.1186/s12939-016-0493-7

**Published:** 2016-12-09

**Authors:** Kumanan Rasanathan, Theresa Diaz

**Affiliations:** Health Section, UNICEF, 3 UN Plaza, New York, NY 10017 USA

**Keywords:** Health equity, Social determinants of health, Implementation research, Research agenda, Sustainable development goals

## Abstract

**Background:**

The tremendous increase in knowledge on inequities in health and their drivers in recent decades has not been matched by improvements in health inequities themselves, or by systematic evidence of what works to reduce health inequities. Within health equity research there is a skew towards diagnostic studies in comparison to intervention studies showing evidence of how interventions can reduce disparities.

**Main text:**

The lack of sufficient specific evidence on how to implement specific policies and interventions in specific contexts to reduce health inequities creates policy confusion and partly explains the lack of progress on health inequities. In the field of research on equity in health, the time has come to stop focusing so much energy on prevalence and pathways, and instead shift to proposing and testing solutions. Four promising approaches to do so are implementation research, natural experimental policy studies, research on buy-in by policy-makers to action on health inequities, and geospatial analysis.

**Conclusion:**

The case for action on social determinants and health inequities has well and truly been made. The community of researchers on health equity now need to turn their attention to supporting implementation efforts towards achievements of the Sustainable Development Goals and substantive reductions in health inequities.

## Background

The field of research into health equity is now well established. Almost 40 years have passed since the Declaration of Alma Ata highlighted the importance of action on health inequities [1]. Over 25 years have passed since the seminal works on health equity by Margaret Whitehead and Michael Marmot [2, 3]. And almost 8 years have passed since the Commission on Social Determinants of Health released its findings, including emphasizing the importance of research on health equity [4].

Much has been achieved. In parallel with a resurgence of interest in equity in other spheres, in particular in manifestations of economic inequality, a focus on health inequity is now centrally seen in global and national policy dialogues. The recently adopted Agenda 2030 for Sustainable Development highlighted the importance of addressing equity and of considering targets only achieved if this occurred across different groups within society [5]. The importance of disaggregating data for health outcomes is now well recognized.

At the same time, progress has been mixed. The tremendous increase of knowledge on inequities in health and their drivers has not been matched by improvements in health inequities themselves, or by systematic evidence of what works to reduce health inequities. Indeed, there remains within health equity research a skew towards diagnostic studies which describe health inequities and their causes, in comparison to intervention studies which show evidence of how interventions can reduce disparities. This mismatch poses the question whether even greater attention is needed to the long-standing call to shift the focus of research on health inequities from the problem to the “solution space” [6].

## Main text

Major new studies on the prevalence and causes of health inequities remain important and useful. For example, there have been recent publications on the social determinants of young people’s health in Europe and North America and on increasing health disparities by income and decreasing disparities by race in the United States [7–9]. But, increasingly, such studies highlight the indecision on policy implications to reduce health disparities in the absence of evidence for interventions. For example, the aforementioned study on disparities by income found lesser health disparities in New York, a city with very high income inequality, than in other U.S. cities with lower income inequality. Income inequality is seen as a major driver of the social gradient in health, yet this study’s results has some speculating that too much emphasis is placed on attention to this determinant [10].

In the absence of greater evidence on which specific interventions or policies reduce health inequities, and in which specific contexts, such confusion is understandable. The classical texts on social determinants of health and health inequities have focused on articulating the prevalence of disparities and the pathways that explain them. But the lack of understanding or motivation among decision-makers about which policies to implement, and the lack of progress on reducing health inequities, suggests that the time has come to stop focusing so much energy on prevalence and pathways, and instead shift to proposing and testing solutions.

Part of the reason that research on health equity has shied away from evaluating interventions is the methodological difficulty in doing so. By definition, the social determinants which generate health inequities are far-reaching across all of society, and single interventions or policies cannot necessarily be expected to address all of them. Mainstays of intervention research such as randomized trials are also often not feasible. But these challenges are not unique to research on health inequities, so new models of research are required to catalyse greater action on social determinants. In particular, four promising and neglected approaches in health equity research can be identified.

First, an area that merits particular attention is implementation research – “scientific inquiry into questions concerning implementation—the act of carrying an intention into effect, which in health research can be policies, programmes, or individual practices (collectively called interventions)” [11]. Such research is being increasingly employed in health systems research. Its focus is particularly on how to implement interventions and how to overcome barriers to implementation. Implementation research tends to be context-specific and aims to specifically address the concerns of implementers and policymakers. It is less concerned with producing generalizable estimates of the effect size of interventions.

Given the extensive policy prescriptions available for reducing health inequities, for example from the report of the Commission on Social Determinants of Health, implementation research can play an important role in assisting policy makers and implementers to translate these high-level prescriptions into implementable and evaluable interventions and policies, tailored to their own contexts. For example, the Commission recommended that governments provide universal coverage of early childhood development programmes. While there are good examples of early childhood development programmes in many countries, these are mostly in upper-middle or high-income settings, and the empirical impact of these programmes on health inequities is unclear. Implementation research could help to unpack what is required for early childhood development programmes in low- and middle-income countries, and also how their impact to reduce inequities can be maximized in specific contexts.

Second, there is scope for greater use of political epidemiology approaches that evaluate the impact of specific policy changes. Such studies “analyze the introduction of new legislation or a policy change as a natural experiment, attempting to identify the causal effect of change in the exposure on change in the outcome, using such causal inferential methods as instrumental variable, fixed effects or discontinuity regression analysis” [12]. The aim of these approaches are to satisfy the demand of decision-makers for evidence for policies and interventions on health disparities. Taking the case of the aforementioned differences in income inequality and health disparities in the United States, studies to evaluate the contribution of different policies on health disparities between, for example, New York and Detroit, might provide useful intervention and policy evidence that could be used to convince policymakers.

Third, a further fertile research area is on why the increase of evidence on health disparities and the role of social determinants has not had a greater effect on policy and practice, predominantly using qualitative methods. There are some pioneering studies on this question [13], but much more insight is required, particularly with regard to policymakers in low- and middle-income countries.

Policymaking is not merely a technocratic process. Researchers, and the broader community working towards health equity, need to better understand challenges in the political economy in countries to implementing effective policies to reduce health inequities. In recent years in the field of child health, UNICEF has undertaken analyses that show that prioritization of the worst off children is not only essential for the moral imperative to reduce health inequities, but that it is also the most efficient means of using resources to achieve child health targets [14]. Further analysis would be useful to understand whether these types of arguments sway policymakers and influence the allocation of resources, or, if not, what other types of evidence might.

Fourth and finally, new innovations in geospatial mapping and analysis can help to uncover where to best prioritize new resources for marginalized communities, and also to better understand the relationships between different policy interventions on the social determinants. Returning again to the example of less than expected health disparities in New York, such analysis could help to unpack the effect of improved access to transport and green spaces on inequities.

## Conclusion

The 2030 Agenda for Sustainable Development and the accompanying Sustainable Development Goals (SDGs) provide a major opportunity to turn the tide on health inequities. To grasp this opportunity, research on equity in health needs reorientation towards a policy and implementation agenda, as well towards building capacity in monitoring inequities in all countries, focusing not just on health outcomes but linked to the health-related indicators across SDG goals and sectors. But attention to monitoring of health inequities should be a secondary aim to greater evidence for and guidance to implementation.

The case for action on social determinants and health inequities has well and truly been made. The community of researchers on health equity now need to turn their attention to supporting implementation efforts towards achievements of the SDGs. Greater attention to implementation research, natural experimental policy studies, research on buy-in by policy-makers to action on health inequities, and geospatial analysis are all opportunities to do so.

## Abbreviation

SDGs: Sustainable development goals
